# Supplementary material for: Multimodal imaging and functional analysis of the chick NMDA retinal damage model
Source: PLoS One. 2021 Sep 7;16(9):e0257148. doi: 10.1371/journal.pone.0257148 (PMC8423281; doi:10.1371/journal.pone.0257148)
Supplement: S1 File — (ZIP) [file pone.0257148.s004.zip › SD-OCT/C200727/016/D15-PWk3/C200727-016_OS_V_14X14_0_REGAVG0000053/Manual_PreDefineFiveByFiveGridRectangularRetina_1.pdf]

Five By Five Grid Template Report

**Patient ID**	C200727	**Name**	See InVivoVue
**Exam Date**	See InVivoVue	**Exam Time**	See InVivoVue
**Eye**	OS	**Averaged**	N/A
**Depth (mm)**	1.65	**Depth Samples**	1024
**Pitch (um/pix)**	35.000 x 35.000 x 1.608	**Refractive Index**	See InVivoVue
**Scan Type**	Rectangular 14.000mm x 14.000mm x 1.647mm , 400 x 400 x 1024		

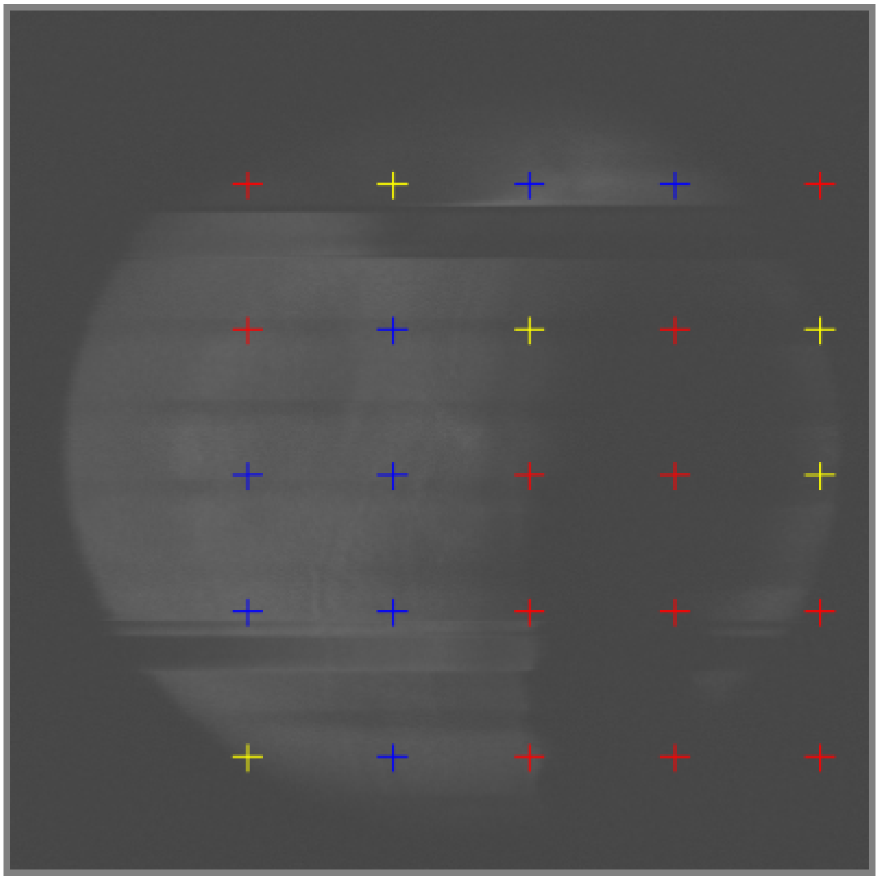

Thickness Metrics

**Analysis**	**Average(mm)**	**Minimum(mm)**	**Maximum(mm)**	**STDEV(mm)**
**IRNFL - RPE**	0.2774	0.2577	0.2971	0.0135
**IRNFL - ORNFL**	0.0465	0.0144	0.0749	0.0211
**ORNFL - OPL**	0.1558	0.1248	0.1885	0.0236
**OPL - ETPRS**	0.0552	0.0468	0.0637	0.0049
